# Factors that influence married/partnered women’s decisions to use contraception in Zambia

**DOI:** 10.3389/fgwh.2023.1157097

**Published:** 2024-02-21

**Authors:** Chilochibi Chiziba, Mwimba Chewe, Peter Hangoma

**Affiliations:** ^1^ Department of Health Policy and Management, School of Public Health, University of Zambia, Lusaka, Zambia; ^2^ Chr. Michelsen Institute (CMI), Bergen, Norway; ^3^ Bergen Center for Ethics and Priority Setting in Health (BCEPS), University of Bergen, Bergen, Norway

**Keywords:** women, contraception use, decision-making, Zambia, household

## Abstract

According to the Demographic and Health Surveys (DHSs), Zambia has shown an increasing trend in the percentage of married women using contraceptives in the last three decades. As of 2018, this percentage increased from 34.2% in 2001 to 40.8% in 2007 and from 45% in 2013 to 48% in 2018. Despite the increasing trend in contraceptive use, the unmet needs remain relatively high. The low percentage of contraception use translates into 20% of women of reproductive age who are either married/partnered and want to stop or delay childbearing but are not using contraception. This study analyzed factors other than availability that influence women’s ability to make or influence the decision to use contraception using logistic regression using data from the Zambia 2013/2014 and 2018 DHSs. Furthermore, adjusted odds ratios and predicted probabilities were estimated using the fitted logistic regression. Data on 8,335 women were analyzed, and 13.7% (*n* = 1,145) had their husband as the sole decision maker for contraception use, while 86.3% (*n* = 7,189) made the decisions or participated in making the decision. Contrary to most literature, those with primary or secondary school education were less likely to decide than those without education. The data also associate women who contribute to daily household decisions to having a say in deciding to use contraception. Lastly, women using reversible contraception methods, other methods, hormonal methods, and fertility awareness were associated with less likelihood to decide on using contraceptives than those using barrier methods. Women with lower household decision-making powers are less likely to make or influence decisions to use contraception. Consequently, there is a need to prioritize such women in interventions aimed at increasing contraception use decision-making. Furthermore, more studies are required to investigate why uneducated women in Zambia are more likely to choose contraception. Also, the vast odds ratio difference between all other methods compared to barrier methods (condoms) indicates underlying factors that play a role, which warrants further studies.

## Background

According to the Zambia Demographic and Health Surveys (DHSs), Zambia has seen an increasing trend in married women using contraceptives in the last three decades. As of 2018, this percentage increased from 34.2% in 2001 to 40.8% in 2007 and from 45% in 2013 to 48% in 2018. The low percentage of contraception use translates into 20% of women of reproductive age who are either married or in a union who want to stop or delay childbearing and are still not using contraception (1).

Despite the increasing trend in contraceptive use and considerable investments by the Zambian Government complemented by several non-governmental organizations in demand generation efforts, unmet need for contraceptives remain relatively high at 20% (2). Several resources have been invested in the family planning space, including access to family planning, community engagement, expanded dialog on family planning, and improved coordination to increase contraception supply. However, at an individual level, women may demand and collect contraceptives from the facility, but the decision to use them is not always guaranteed (3). Consequently, the unguaranteed use exacerbates the delays in bridging the unmet needs gap, which necessitates looking at factors beyond the availability of contraceptives and other family planning resources that influence women’s ability to decide or influence the decision to use contraception.

To complement the government’s efforts in the family planning space, several studies focusing on interpersonal communication, types of contraception, and supply of contraceptives have been conducted in Zambia (4). However, these studies have mainly focused on supply and demand for contraceptives without drilling down to microlevel dynamics between the men and women before they decide to use or not use contraception (4). This study, consequently, looks beyond the availability and access to contraceptives. Instead, it focuses on woman’s ability to decide independently or discuss using modern contraception in a household with their partner (5).

The study analyzes how individuals’ choices to use contraceptives depend critically on their perception of the actions of their partners because of that choice. The contraception use decision dynamics occur in a bedroom and are motivated by individual preferences and satisfaction maximization, which are influenced by various factors and a train of recursive bargaining among couples. The recursive bargain conforms to psychological reasoning explained by game theory (6, 7). In the man and woman contraception decision-making scenario, the outcome may depend on their background (6). For example, higher education attainment may lead to increased income and greater independence for a woman (8). With higher income, women may possess relatively higher bargaining power. In extreme cases, if a woman’s fertility preferences are threatened by a man unwilling to let her decide, she can credibly threaten to leave the man. On the other hand, the man may not consider the woman’s threat credible if she does not participate in the daily household decisions as he expects to be allowed to “not cooperate”. After all, the outcome will be better for both. The woman may feel compelled to keep silent about her contraception preferences in order to avoid conflict, abuse, or separation (6).

Having established the potential existence of intrahousehold barriers to contraception use decision-making, the study, therefore, analyzes factors other than availability that influence married (officially married or living with a partner) women’s ability to decide or influence the decision to use contraception. The results of this study may contribute to the knowledge needed to bridge the unmet needs gap from an individual level. Addressing the contraception issue from the perspective that this study is taking is essential; without such studies, investment efforts will continue to be relatively ineffective in addressing high unmet needs for family planning (2).

This study will help the public health field and policymakers understand the factors other than availability that influence the dynamics behind women’s ability to decide or influence the decision to use contraceptives. The study’s focus on women’s ability to decide to use contraception provides a direct link to the family planning indicators, such as unmet needs for family planning (4, 9).

Furthermore, with the limited availability of resources, the study findings may inform intervention prioritization by looking at how factors are associated with women’s decisions to use contraception. That way, the intervention focus can be channeled toward dealing with triggers and not symptoms of the lack of contraception use.

The study assumes that a reduction in unmet needs may also reduce other health complications and socioeconomic burdens that come with not utilizing contraception. The analysis also assumes that its findings will enable policymakers to add programs and initiatives in line with the study findings to promote reducing unmet family planning needs (10).

## Methodology

### Model specification

A model aimed to capture the factors that influence women’s ability to make or influence the decision to use contraception was contracted. Since the ability to make or influence the decision to use contraception has two potential outcomes (yes or no), the relationship was translated into a decision influencer equation that modeled women’s decision to use contraceptives in a marriage/union as a function of their freedom to make household decisions and to have free time, and their socioeconomic (employment status, education, etc.) status, fertility preferences, husband's desire for children, choice of contraceptive type, and involvement in daily household decisions, as illustrated by Equation 1. Since the possible outcomes are binary, a logistic regression model was utilized. We chose a binary logistic regression model because we want to estimate women’s probabilities of being able to make or influence the decision to use contraception (11). Several models were fit with varying covariate combinations or processing approaches during the model fitting process. However, the model with the lowest Akaike information criterion (AIC) was selected as the final model. Furthermore, after fitting the final logistic regression, a receiver operating characteristic curve (ROC) was constructed to validate the model’s prediction performance (11):

(1) $$\begin{array}{c} y_{i}=\left\{\begin{array}{c} 1\;\mathrm{if}\;\mathrm{woman}\;\mathrm{is}\;\mathrm{the}\;\mathrm{decision}\;\mathrm{maker}\;\mathrm{or}\;\mathrm{influencer}\;\mathrm{for}\;\mathrm{using}/\\ \mathrm{not}\;\mathrm{using}\;\mathrm{contraception}\\ 0\;\mathrm{if}\;\mathrm{man}\;\mathrm{is}\;\mathrm{the}\;\mathrm{decision}\;\mathrm{maker}\;\mathrm{for}\;\mathrm{using}\;/\mathrm{not}\\ \mathrm{using}\;\mathrm{contraception} \end{array}\right\}\\ \log\left(\frac{\mathrm{Pr}(y=1|X_{i})}{\mathrm{Pr}(y=0|X_{i})}\right)=\alpha +X_{i}\beta \end{array}$$

In this equation, y*_i_* represents the options available to a woman in a “contraception decision-making bargain,” X*_i_* is a matrix notation representing all model predictors selected based on the literature and data availability, α represents the model intercept, *i* represents women, and β represents the coefficients for all the regressors (11). Similar to other studies, for cases where the decision was made jointly, this study assumed the woman made the decision (3).

### Data, sample size, and sampling methods

The 2013/2014 and 2018 Zambia DH surveys provided data for all variables outlined in Table 1, which were selected based on the literature, public availability, and representativeness of DHS data. The DHS is a systematic multistage survey by the DHS Program that aims to collect accurate, nationally representative health and population data in developing countries. The 2013/2014 and 2018 Zambia DHSs used the 2010 Zambia Census of Population and Housing as its sampling frame, comprising stratification by provinces, districts, constituencies, wards, and enumeration areas (EAs). Each EA comprised approximately 130 and 110 households in the 2013/2014 and 2018 surveys, respectively. The EA list was updated for household changes since the 2010 census and used for sampling. The two-stage stratified approach involved selecting clusters of EAs proportional to their size, followed by systematic household sampling. Each survey collected data from 18,052 and 13,625 households in 2013/2014 and 2018, respectively, representing national, urban, rural, and provincial levels. For this study, only women aged 15–49 years who were “married” or “living with a partner” were included, totaling 8,335 participants (1, 12).

**Table 1:** Covariates explored and their definitions.

Variable ID	Variable definition	Response	Scale of measurement
	Dependent variable		
z	Decision maker for using/not using contraception	Woman/Joint Man	Binary
	Independent variables		
X_1_	Decision index		Proportion
X_2_	Current contraceptive method:	Yes	Nominal
	Subquestions include the following:	No	
	i. Barrier methods		
	ii. Fertility awareness		
	iii. Hormonal methods		
	iv. Other methods		
	v. Permanent		
	vi. Long-acting reversible		
X_3_	Highest education level	A. No education	
		B. Primary	
		C. Secondary	Ordinal
		D. Higher	
X_4_	Employment status	A. All year	Nominal
		B. Seasonal	
		C. Not employed	
X_5_	Wealth index	A. Richest	Ordinal
		B. Richer	
		C. Middle	
		D. Poor	
		E. Poorest	
X_6_	Type of residence	A. Rural	nominal
		B. Urban	
X_7_	Marital status	A. Married	
		B. Non-marriage union	
X_8_	Age	15–49 years	Integer
X_9_	Fertility preference	A. Have another	Nominal
		B. No more	
		C. Undecided	
		D. Sterilized	
X_10_	Husband’s desire for children	A. Both want	Nominal
		B. Husband wants more	
		C. Do not know	
X_11_	Concealing of contraception	A. Yes	Binary
		B. No	
X_12_	Woman earning more	A. Yes	Binary
		B. No	
X_13_	Interview year	A. 2013	Nominal
		B. 2014	
		C. 2018	

### Data processing and analysis

The study used R software to analyze variables of interest using the “survey” package. Before performing any data analysis, the study considered survey weights for external validity purposes. The analyses performed include descriptive and multivariate regression. The descriptive analysis described the characteristics of the study participants and variable outputs; the multivariate logistic regressions modeled who decided to use contraception. Furthermore, due to the miniature representation of the 2013 and 2019 observations, the observations were regarded as 2014 and 2018, respectively, during the model fitting. Doing so ensured that the very few observations for the years 2013 and 2019 were not subjected to too many variables, thereby preventing sample size bias.

### Processing the dependent variable

The DHS captures the decision maker variable to use contraception as a non-binary variable. Thus, the responses captured are woman, man, and joint. To meet the objective of this study, we created a “woman decision maker/influencer to use contraception” binary variable. For this variable, observations were assigned a 1 if the decision to use contraception was made by a woman or jointly; otherwise, a 0 was assigned. As mentioned earlier, this approach is common in studies and fields related to women’s contraception use decision-making (13).

### Calculating the decision index

Literature highlights that women’s ability to influence certain decisions is furthered when they are allowed to or can make daily household decisions (14). The daily household decisions capture who decides when to seek treatment when a child is sick, spending of household earnings, visitation of relatives, and food consumed (1). To capture the role played by daily household decision-making in influencing the decision to use contraception, this study adopted a decision index based on the DHS decision variables in Table 1. However, the study only utilized six decisions (2–7) due to the non-availability of responses in both 2013/2014 and 2018 DHSs for the first two decision questions. The index was formulated as a proportion by dividing the total number of decisions the woman made by the total household decisions considered in this study (15).

### Processing the contraception method variable

As highlighted in Table 1, the contraceptive method variable captures a multitude of contraceptives that people use. However, the methods are relatively too many to place in a model for this study to achieve its objective. Therefore, the various methods were grouped into six common categories used in several studies, such as that by (16). These categories include barrier, fertility awareness, hormonal, permanent, long-acting reversible, and other methods (16).

### Ethical considerations

This study used deidentified, publicly available data from the 2013/2014 and 2018 DHSs: https://dhsprogram.com/Countries/Country-Main.cfm?ctry_id=47&c=Zambia.

Ethical clearance for the study was obtained from the Institutional Review Boards (IRBs) of the University of Zambia (UNZA). Biomedical Research Ethics Committee (UNZABREC). The survey ensured compliance with international ethical standards of informed consent, voluntary participation, and privacy and confidentiality. The dataset was requested, and a letter of data authorization was received from ICF International through the DHS Program. The dataset and more details regarding the ethical standards of the DHS data are available at: https://www.dhsprogram.com/What-We-Do/Protecting-the-Privacy-of-DHS-Survey-Respondents.cfm.

### Limitations of the study

The study lacks the ability to establish causality due to the cross-sectional nature of the data used. Model-wise, the logistic regression model may not appropriately capture non-linear and multiple decision boundaries, thereby limiting the study to capture more complex links that may exist in the data. Furthermore, the study did not add all potential factors, such as psychological or control for all potential confounders, as much as the theory may have dictated due to the limited availability of data captured by the DHS.

## Results

### Descriptive statistics

#### Background characteristics of the study participants

Table 2 shows the overall sample of 8,335 women stratified by the decision maker to use contraception. Of all the participants, 13.7% (*n* = 1,145) had their husbands as the sole decision-makers, while 86.3% (*n* = 7,189) made the decisions or participated in making the decision. Each category is accompanied by a percentage that sums vertically. Age group-wise, most participants were aged between 25 and 29 years, representing 23% (*n* = 1,897) overall and 25% (284) of women whose decision to use contraception were made by their partners. Also, the same age group comprised most women decision-makers, representing 22% (*n* = 1,613). Furthermore, the study findings showed that 99% (*n* = 8,273) of the participants characterized their union as a marriage.

**Table 2:** Demographic and socioeconomic characteristics of the study participants.

Characteristics	Overall, *N* = 8,335	Husband/partner is the decision maker, *N* = 1,145	Woman is the decision maker, *N* = 7,190
Age group			
15–19	380 (4.6%)	71 (6.2%)	309 (4.3%)
20–24	1,440 (17%)	193 (17%)	1,247 (17%)
25–29	1,897 (23%)	284 (25%)	1,613 (22%)
30–34	1,739 (21%)	230 (20%)	1,509 (21%)
35–39	1,416 (17%)	188 (16%)	1,228 (17%)
40–44	983 (12%)	125 (11%)	858 (12%)
45–49	480 (5.8%)	54 (4.7%)	426 (5.9%)
Current marital status			
Married	8,273 (99%)	1,133 (99%)	7,140 (99%)
Living with the partner	62 (0.7%)	12 (1.0%)	50 (0.7%)
Type of place of residence			
Urban	3,601 (43%)	478 (42%)	3,123 (43%)
Rural	4,734 (57%)	667 (58%)	4,067 (57%)
Highest educational level			
No education	694 (8.3%)	93 (8.1%)	601 (8.4%)
Primary	4,317 (52%)	620 (54%)	3,697 (51%)
Secondary	2,837 (34%)	392 (34%)	2,445 (34%)
Higher	485 (5.8%)	40 (3.5%)	445 (6.2%)
Wealth index			
Poorest	1,410 (17%)	197 (17%)	1,213 (17%)
Poorer	1,621 (19%)	248 (22%)	1,373 (19%)
Middle	1,855 (22%)	246 (21%)	1,609 (22%)
Richer	1,758 (21%)	244 (21%)	1,514 (21%)
Richest	1,691 (20%)	210 (18%)	1,481 (21%)
DHS interview year			
2013	2,804 (34%)	501 (44%)	2,303 (32%)
2014	1,790 (21%)	250 (22%)	1,540 (21%)
2018	3,644 (44%)	389 (34%)	3,255 (45%)
2019	97 (1.2%)	5 (0.4%)	92 (1.3%)

Socioeconomically, 57% (*n* = 4,734) resided in rural areas and 52% (*n* = 4,317) of all participants listed primary education as their educational attainment. Higher education had the least respondents, representing 5.8% (*n* = 485). Furthermore, most respondents belonged to the middle class on the wealth index, representing 22% (*n* = 1,855) compared with the poorest contributing the least respondents, of 17% (1,410).

Finally, 3,644 of 8,335 observations in the study were interviewed in 2018 in the 2018 DHS, representing the majority at 44% (*n* = 3,644), followed by 2013, 2014, and 2019 as the least, representing 1.2% (*n* = 97).

#### Women’s household decision-making index by the contraception use decision maker

**Figure 1:**
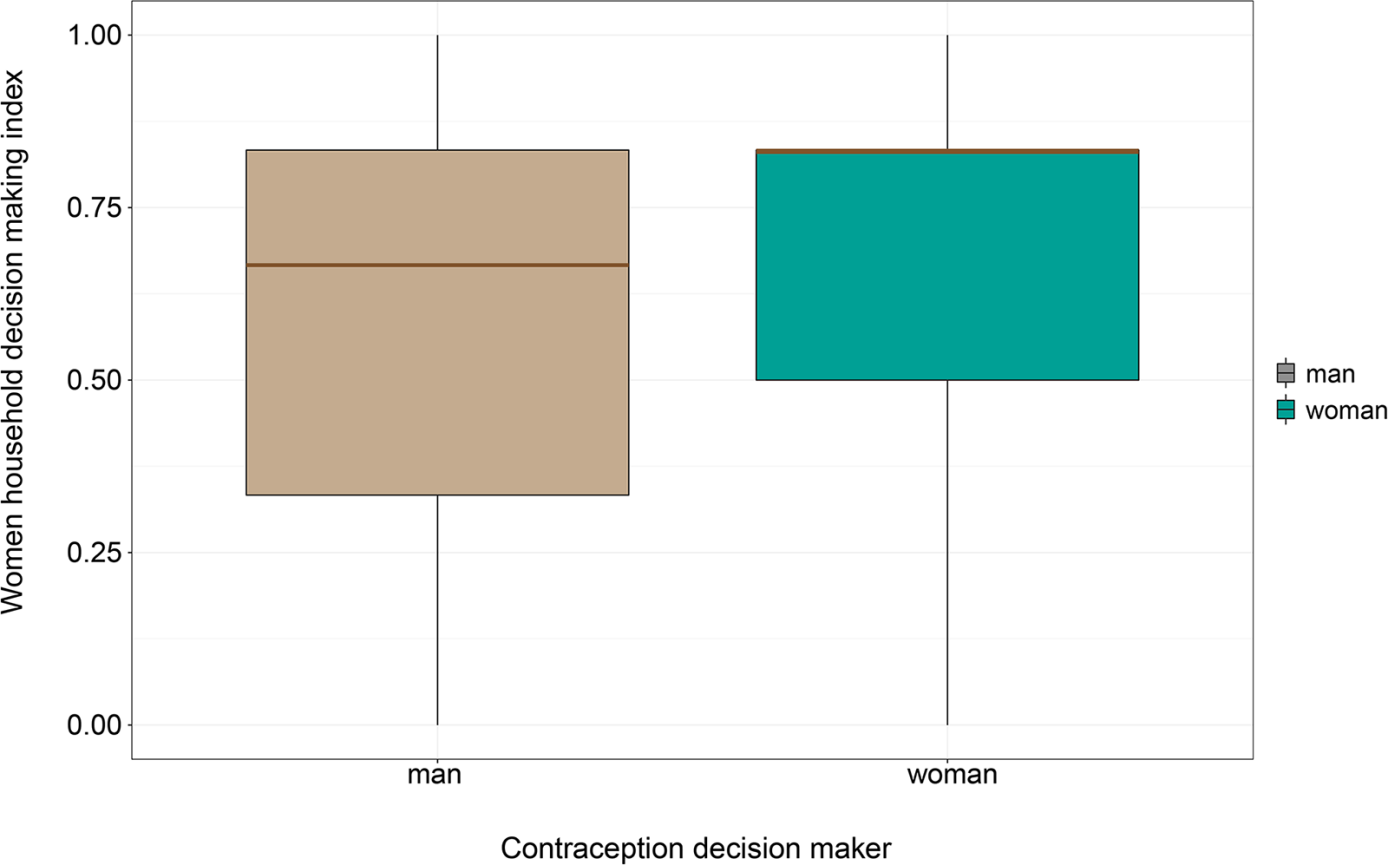
Women’s household decision-making index by the contraception decision maker.

Figure 1 shows the dispersion of observations of women’s household decision-making index across who decides to use contraception. The gray boxplot shows the distribution of observations for women whose partners decided to use contraception. The orange box plot shows observations of women who made or influenced the decision to use contraception. The figure suggests that the index is right-skewed for both decision-makers. However, the median value (green line) for women whose decisions are made by men is about 0.65, with the first quintile of about 0.35.

On the other hand, the median value for female decision-makers is about 0.8, with the first quantile of about 0.50. Also, the third quantile for female and male decision-makers is about 0.8. This suggests that most women with a say in contraception use have a higher decision-making index than those who do not have a say in contraception use.

#### Distribution of husband/partner’s preferences and contraception decision-making

Table 3 suggests that most respondents wanted the same number of children as their husband/partner, with an overall 57% (*n* = 3,534) representation. This preference was followed by husbands wanting more, with a 32% (*n* = 2003) representation, and 10% (*n* = 627) whose husbands wanted fewer.

**Table 3:** Distribution of husband/partner’s preferences and contraception decision-making.

Characteristics	Overall, *N* = 8,335	Husband/partner is the decision maker, *N* = 1,145	Woman is the decision maker, *N* = 7,190
Husband’s desire for children			
Both want the same	3,534 (57%)	479 (56%)	3,055 (58%)
Husband wants more	2,003 (32%)	285 (33%)	1,718 (32%)
Husband wants fewer	627 (10%)	90 (11%)	537 (10%)
Unknown	2,170	291	1,879

#### Distribution of women’s preferences and their ability to make or influence the decision to use contraception

Table 4 shows the distribution of woman’s desire for children. The tables show that most women (44%, i.e., *n* = 3,653) wanted more children after 2 years. Also, of all women who responded that their husband/partner decided to use contraception, 44% (*n* = 502) of them wanted children after 2 years. These were followed by those who “wanted no more,” with a 35% (*n* = 398) representation, those who “wanted within 2 years,” with an 8.4% (*n* = 96) representation, “sterilized (respondent or partner),” with a 5.6% (*n* = 64) representation, “wants, unsure timing,” representing 2.9% (*n* = 33), and “undecided,” with a 4.2% (*n* = 48) representation. Lastly, those declared infecund had the least representation of 0.3 (*n* = 3).

**Table 4:** Distribution of women’s preferences and married women’s ability to influence the decision to use contraception.

Characteristics	Overall, *N* = 8,335	Husband/partner is the decision maker, *N* = 1,145	Woman is the decision maker, *N* = 7,190
Women’s fertility preferences			
Wants within 2 years	745 (9.0%)	96 (8.4%)	649 (9.0%)
Wants after 2 + years	3,653 (44%)	502 (44%)	3,151 (44%)
Wants, unsure timing	162 (1.9%)	33 (2.9%)	129 (1.8%)
Undecided	342 (4.1%)	48 (4.2%)	294 (4.1%)
Wants no more	3,086 (37%)	398 (35%)	2,688 (37%)
Sterilized (respondent or partner)	321 (3.9%)	64 (5.6%)	257 (3.6%)
Declared infecund	14 (0.2%)	3 (0.3%)	11 (0.2%)

In total, 44% (*n* = 3,151) of women who decided to use contraception also wanted children after 2 years and those declared infecund had a minor representation of 0.2% (*n* = 11). The data suggest that most respondents in both categories wanted to have children after 2 years.

#### Contraceptive type and married women’s ability to make or influence the decision to use contraception

Table 5 shows different contraceptives that women mentioned as the contraception method they used during the survey, disaggregated by the person who decided to use them. Barrier methods in this study refer to contraceptives, including condoms and female condoms. This category constituted about 7.2% (*n* = 601) of all respondents. According to the findings, 1.4% of respondents practiced fertility awareness (individuals following standard days and periodic abstinence). Also, the data suggested that most women who used contraception used hormonal-related contraception, such as pills, injections, and lactational amenorrhea (lam). Collectively, hormonal methods comprised 67% (*n* = 5,548) of the 8,335 total participants. This category also comprised most of the respondents whose husbands/partners made the decision, similar to when the woman decided. The data also suggested that 6.1% (*n* = 512) of the total respondents used “other” methods. Other methods included uncommon methods such as withdrawal methods. Of the 8,335 respondents, 3.9% (*n* = 321) were using permanent contraception. This category includes female and male sterilization. Finally, 15% (*n* = 1,239) of the respondents stated they used long-lasting reversible contraception, including methods such as intrauterine devices (IUDs) and implants (Norplant).

**Table 5:** Contraceptive types by the decision maker to use contraception.

Characteristics	Overall, *N* = 8,335	Husband/partner is the decision maker, *N* = 1,145	Woman is the decision maker, *N* = 7,190
Contraception method			
Barrier methods	601 (7.2%)	93 (8.1%)	508 (7.1%)
Fertility awareness	113 (1.4%)	12 (1.0%)	101 (1.4%)
Hormonal	5,548 (67%)	729 (64%)	4,819 (67%)
Other	512 (6.1%)	102 (8.9%)	410 (5.7%)
Permanent	321 (3.9%)	64 (5.6%)	257 (3.6%)
Long-acting reversible	1,239 (15%)	145 (13%)	1,094 (15%)

### Statistical model results

#### Results of multivariate logistic regression

Table 6 shows the model coefficients with their respective *p*-values standard error(SE), and t-value.

**Table 6:** Multivariate analysis results.

Variables	Estimate	SE	*t*-value	Pr…t..
(Intercept)	15.379	1.570	9.792	<2 × 10^−16^^a^^,^^b^
Decision index	1.220	0.432	2.825	0.004^b^^,^^c^
Woman’s age	0.029	0.019	1.564	0.118
Education—primary	−2.166	0.766	−2.826	0.004^c^
Education—secondary	−2.157	0.809	−2.664	0.007^b^^,^^c^
Education—higher	−1.700	0.884	−1.922	0.055^d^
Woman earns more	−0.231	0.302	−0.766	0.444
Wealth index—poorer	0.612	0.413	1.479	0.139
Wealth index—middle	0.587	0.416	1.410	0.159
Wealth index—richer	0.840	0.455	1.846	0.065^d^
Wealth index—richest	0.215	0.522	0.411	0.680
Contraception method -fertility awareness	−10.559	0.89802	−11.759	5.14 × 10^−28^
Contraception method -hormonal	−11.555	0.714	−16.174	1.06 × 10^−46^^a^
Contraception method—other	−11.319	1.082	−10.457	4.70 × 10^−23^^a^
Contraception method—reversible	−11.763	0.706	−16.639	8.92 × 10^−49^^a^
Residence—rural	0.094	0.296	0.319	0.749
Marital status—living with partner	−1.633	0.573	−2.850	0.004^c^
Husband child desire—more	−0.439	0.339	−1.295	0.195
Husband child desire—fewer	0.281	0.297	0.947	0.344
Woman child desire—after 2+ years	0.822	0.406	2.022	0.043^e^
Woman child desire—wants no more	0.176	0.355	0.496	0.620
Not concealing contraception	−3.015	1.040	−2.898	0.003^c^
Interview year—2014	0.384	0.242	1.583	0.113

Sample size: 8,335. AIC: 838.7.

Signif. codes: 0.

^a^ 0.001.

^b^ 1.

^c^ 0.01.

^d^ 0.1.

^e^ 0.05.

**Figure 2:**
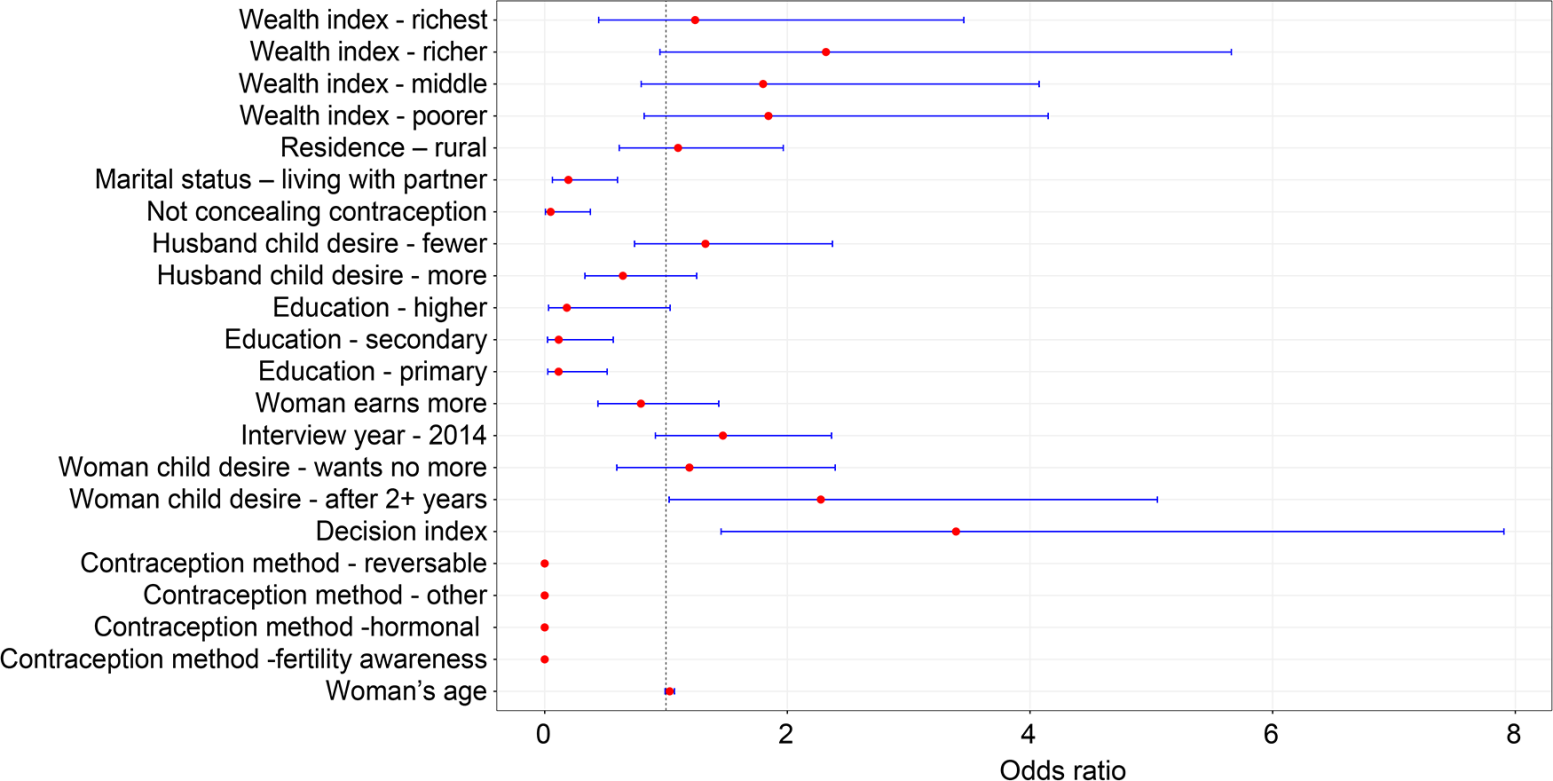
Forest plot of the multivariate logistic regression analysis.

Figure 2 graphically summarizes the odds ratios for the multivariate model. The red dots in the figure represent the odds ratios for all covariates, and the blue bars show their respective confidence intervals. Furthermore, any blue bar crossing the dotted vertical line is not significant.

The results from the multivariate model in Figure 2 suggest that married women were less likely to decide than those “living with a partner” [adjusted odds ratio (aOR) = 0.26, CI = 0.14–0.49]. In the multivariate analysis, women whose husbands knew they were using contraception had significantly lower odds of deciding to use contraception than women who concealed contraception use (aOR = 0.04, CI = 0.02–0.35). Surprisingly, those with primary or secondary school education were less likely to decide than those without education. Furthermore, data suggest that the higher the decision index, the more likely a woman has a say in using contraception (aOR = 3.25, CI = 1.42–7.43).

Lastly, women using reversible contraception methods (aOR = 0.00000816, CI = 0.00000198–0.0000336), other methods (aOR =  0.0000115, CI = 0.00000141–0.0000946), hormonal methods (aOR = 0.0000100, CI = 0.00000243–0.0000413), and fertility awareness (aOR = 0.0000267, CI = 0.00000448–0.000159) were less likely to decide to use contraceptives compared with those using barrier methods.

Validation-wise, the final multivariate model performed reasonably well with an area under the curve (AUC) of 0.67 (11).

#### How daily household decisions affect women’s ability to make or influence the decision to use contraception

**Figure 3:**
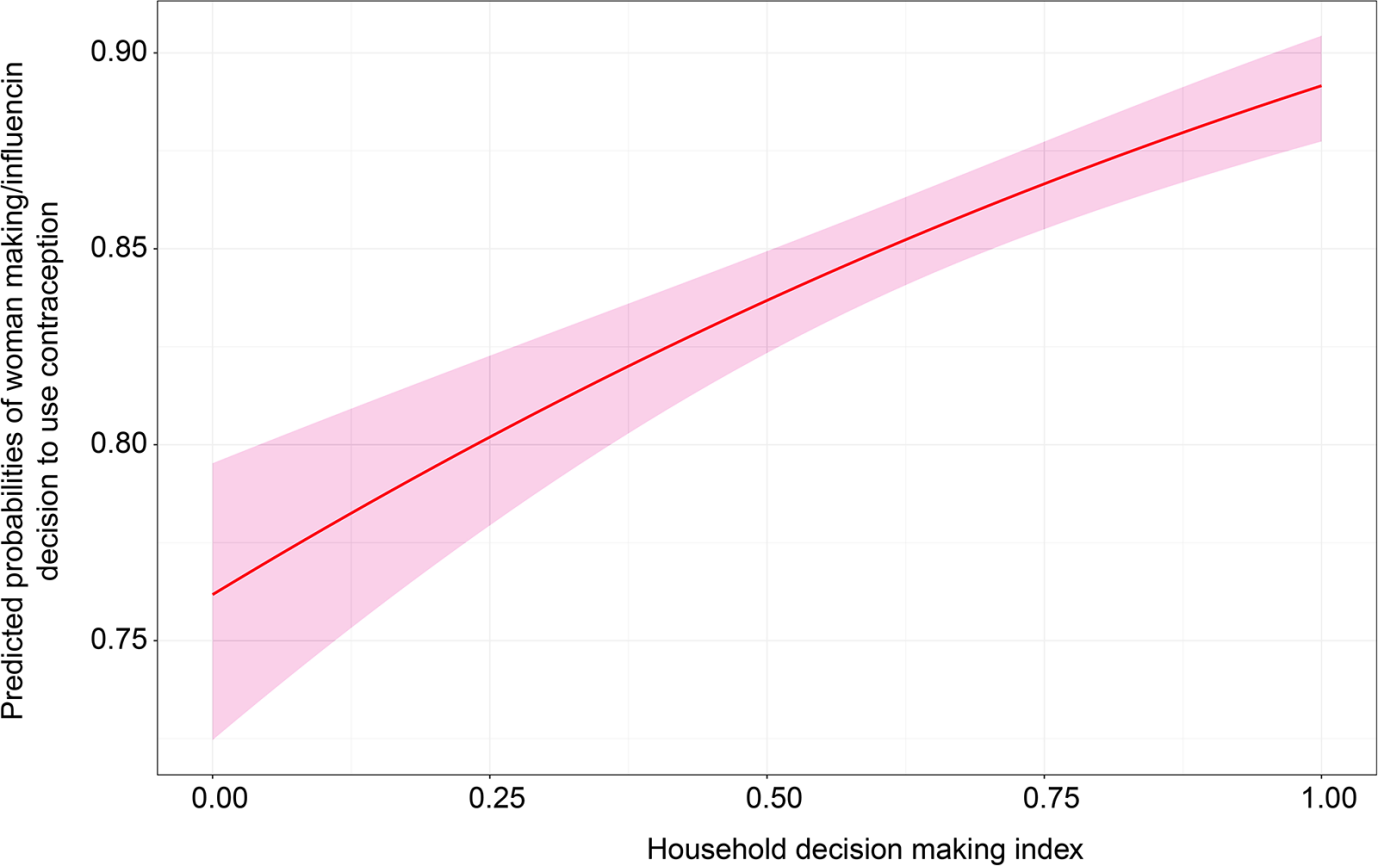
Effect of the decision index on women’s ability to influence the decision to use contraception.

Household deciding powers proxied by the decision index were positively associated with women deciding to use contraception; the effect was pronounced in the multivariate analysis (Figure 3). In the multivariate analysis, women’s probability of influencing contraception use increases with the decision index beginning at 0.74–0.9. This suggests that the higher the woman’s decision index, the higher the probability for her to influence the decision to use contraception.

#### How socioeconomic factors affect women’s ability to make or influence the decision to use contraception

**Figure 4:**
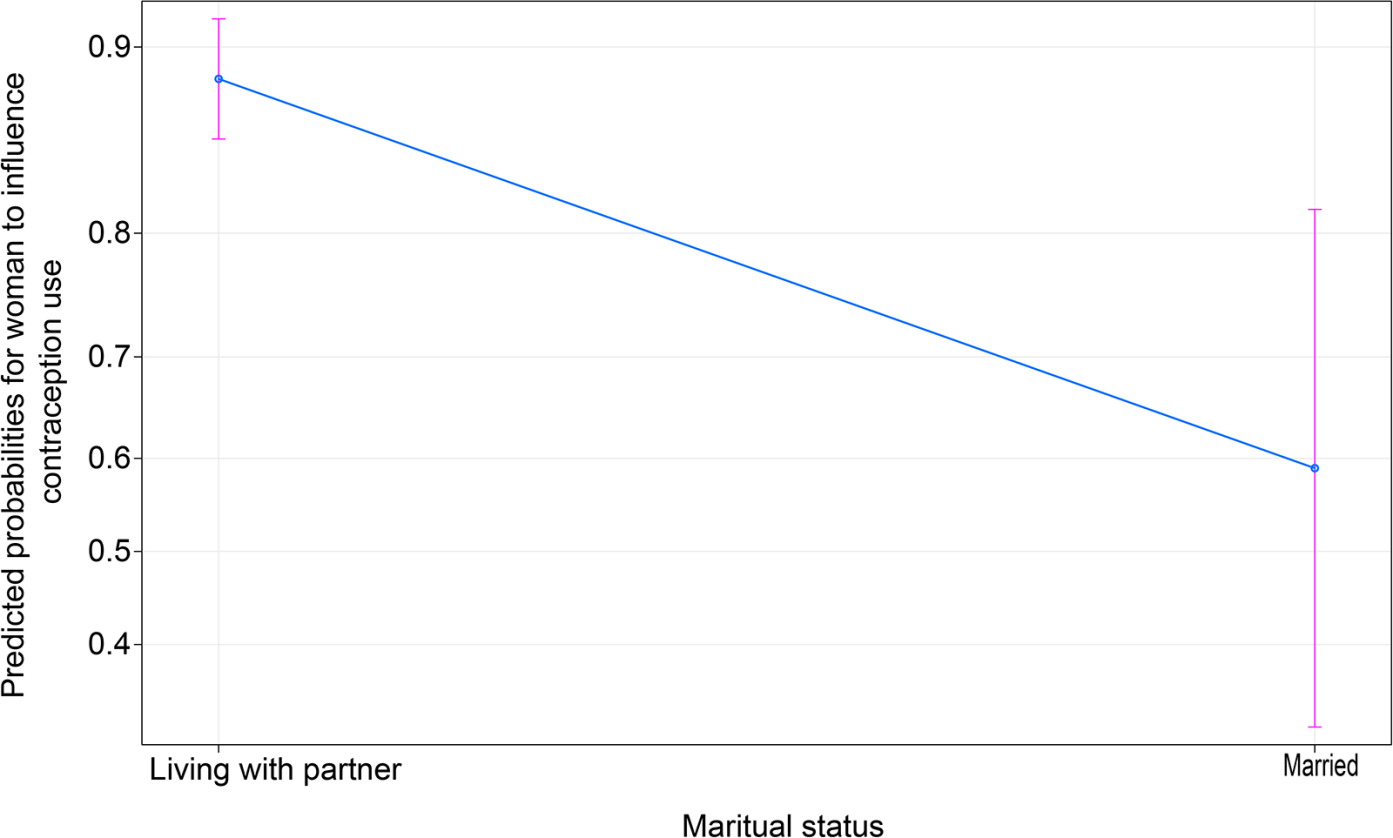
Effect of marital status on women's contraceptives.

Figure 4 shows that living with a partner increases the ability to influence the decision to use contraception being officially married. The figure suggests that women who unofficially live with a partner have about 87% chance of influencing the decision to use contraception compared with those who are “married,” who only have about 59% chance.

**Figure 5:**
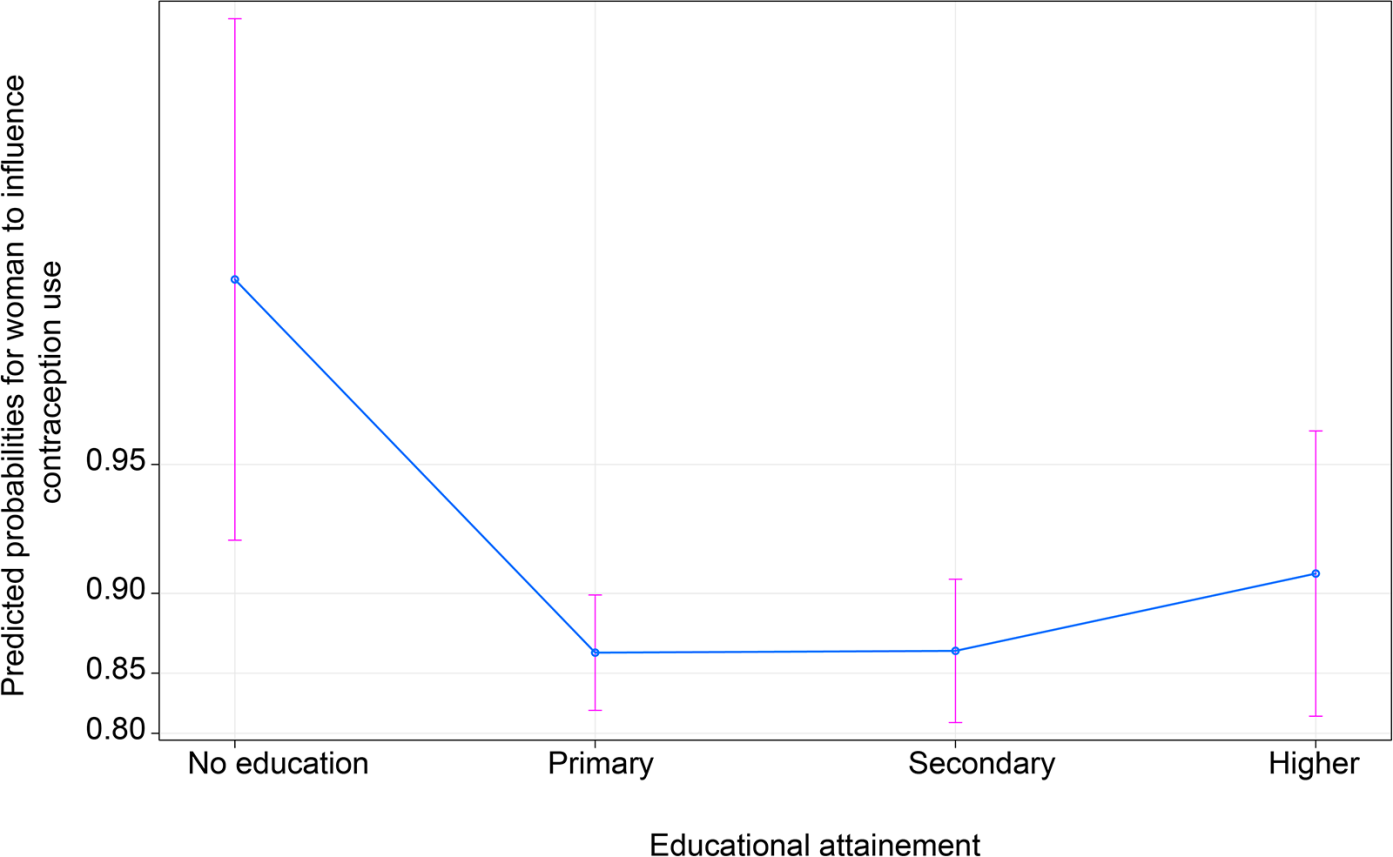
Effect of educational attainment on women’s ability to influence contraception use.

On the other hand, education suggests mixed results from the expectation. In Figure 5, the data indicate that women with no education have a higher probability of influencing contraception use than women with other educational attainments. However, according to the confidence intervals, there seems to be no evidence to suggest superiority between primary, secondary, and higher education. Nevertheless, the predicted probability points for these categories suggest that women with higher education are expected to have a greater influence on contraception use than those with primary and secondary education.

#### How various family planning methods affect women’s ability to make or influence the decision to use contraception

**Figure 6:**
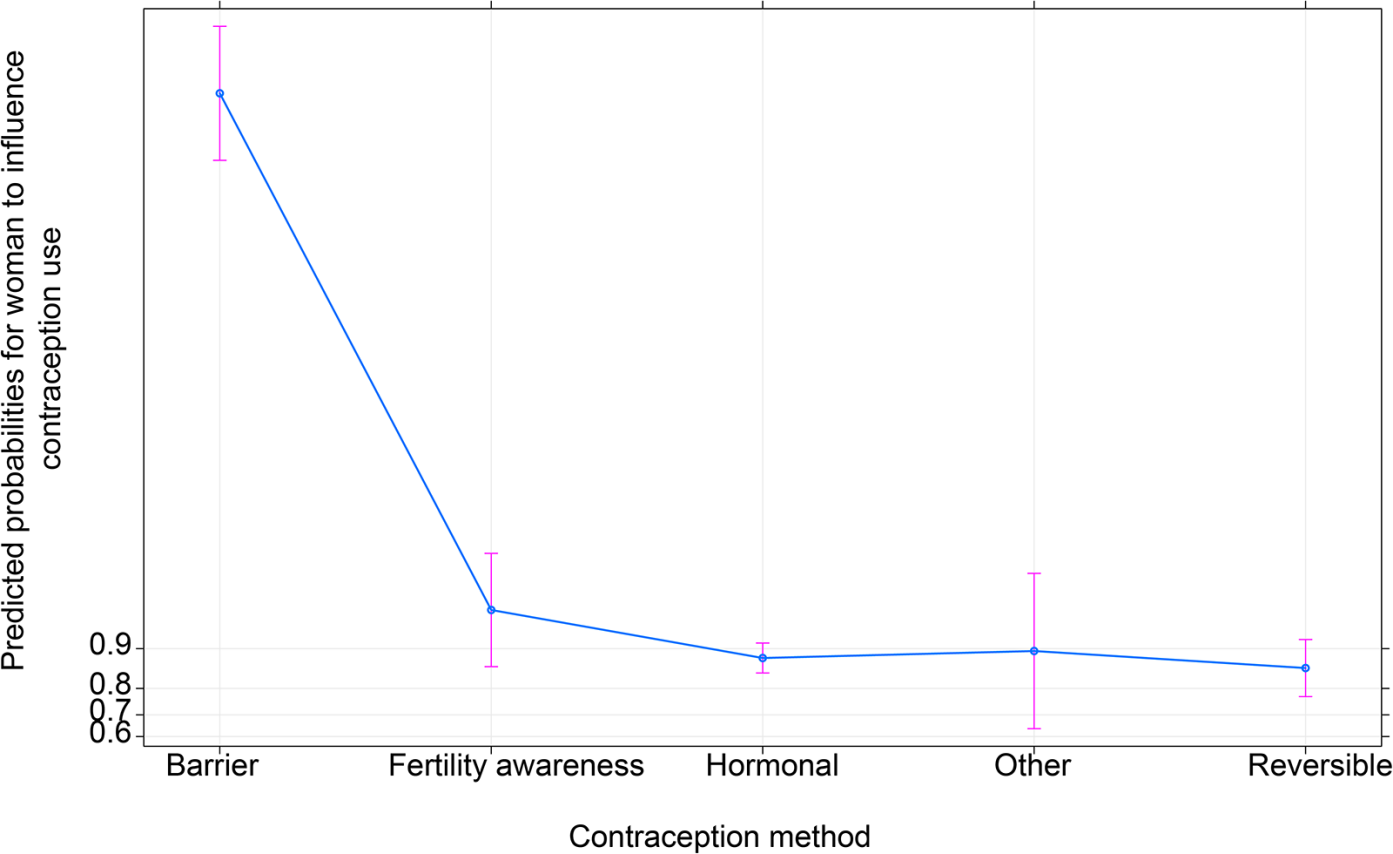
Effect of contraception methods on women’s ability to influence contraception use.

Figure 6 shows the association between predicted probabilities of various family planning methods and women’s ability to decide or influence the decision to use contraception. The figure suggests that women who use barrier methods have the highest likelihood of influencing the decision to use contraception than women who use other contraception methods. Furthermore, differences among the other methods except for barrier methods are insignificant as they lie within the same *y*-axes.

#### Effect of preferences on women’s ability to influence contraception use

The study could not find significant evidence on how fertility preferences and a man’s desired number of children affect the decision to use contraception. Nevertheless, the relationship observed in the logistic regression and chi-square tests were positive for all women’s desired fertility. Furthermore, the data showed opposing results on the partner’s preferences, that is, a negative relationship if the husband wants more and a positive one if the husband desires fewer using “both wanting the same” as a reference variable.

## Discussion

The study analyzed the factors that influence women’s ability to make or influence the decision to use contraception. The factors included household decisions, socioeconomic factors, preferences, and contraception methods. The study used data from 2013/2014 and 2018 DHSs in Zambia. Most of the variables used in the study were categorical, except for age and the formulated decision index.

In support of various studies, the results of this study also suggest that daily household decision-making power, proxied by the decision index, positively affects women’s ability to decide or influence the decision to use contraception. Using the decision index, the study suggests that women with a lower decision index score have lower odds of influencing decisions to use contraception in the household (3, 17). The ability of an individual, particularly a woman, to make decisions on issues regarding the household makes them feel at liberty to in deciding to use contraception. However, deciding alone is not enough; the man must comply with the woman’s decision. If the woman has bargaining power, the man will comply with her contraception decision if he perceives her future household decisions have the ability to benefit or disadvantage him. Therefore, the more decisions the woman makes in the household, the more likely she is to decide to use contraception, and her decision will be considered (18).

Other studies, such as that of OlaOlurum and Hindin (14), also suggest that the number of daily household decisions a woman makes matters regardless of the magnitude of the decision. This is in line with the decision index formulated for this study, which is based on a number of decisions, and all household decisions were weighted equally (14).

Regarding socioeconomic status-related variables, this study hypothesized that education positively influences women’s ability to decide or influence the decision to use contraception after primary school. Unexpectedly, the study found that no education was associated with greater influence in the decision to use contraception. It was observed in Figure 5 that predicted probability values increased with the level of education attainment starting from primary education. However, the confidence intervals for predicted probabilities overlapped, suggesting that the differences in predicted probabilities were insignificant (4).

Nevertheless, other studies, including that of Dadi et al. (19), noted that relatively educated individuals know the benefits of contraception and the implications of not doing so, hence their determination to influence the decision (19). Also OlaOlurum and Hindin (14) stressed that relatively more educated women generally possess a higher likelihood of finding a job and achieving financial independence in the event of separation from the husband that may result from disagreement about household decision-making (14). Based on the literature reviewed in the study, education nevertheless provides the necessary background for understanding how higher women’s socioeconomic factors make their husband/partner comply with their decisions (20). Nonetheless, the conflicting results for education and contraception decision-making for Zambian women warrant further investigation for subsequent studies to ascertain the dynamics that lead to women without education having relatively higher odds of deciding to use contraception.

Furthermore, living unofficially married to a man is associated with having a higher likelihood of using contraception. In the cultural context of Zambia, the expectations of married women likely lead them to choose to let their partner be the sole decision maker as they perceive greater benefit from this arrangement (21).

Although wealth was not significant in this study, one possible reason could be that wealth is a household-level covariate and not individual-based. Therefore, it was insignificant in explaining the woman’s ability to make or influence the decision to use contraception (1). Several other studies, such as that by Madeleine et al. (22), also found weak or insignificant associations between wealth quantiles and the decision to make or influence the decision to use contraception. This may suggest that the overall household makeup may not affect the individual contraception use bargaining dynamics that a couple undergoes (22).

Fertility preference-wise and the husband's desire for children did not show evidence of affecting women’s ability to make or influence the decisions to use contraception. Contrary to this study’s findings, studies in other countries have found significant associations and stressed the need for couples to align preferences at their marriage onset (19).

Finally, the study found that certain contraception methods negatively affect women’s ability to decide or influence the decision to use contraception compared to the barrier method with relatively narrow confidence intervals. Methods such as hormonal and reversible are perceived to have adverse side effects on women; therefore, most women’s ability to decide or influence the decision to use contraception is relatively less positive (16). In addition to the study by Dombola et al. (16), regional and some country-specific studies also highlight similar results, suggesting that this finding may be universal in many other countries (23).

Furthermore, results from this study suggests that the odds of husbands knowing if their partner is using contraception lowers the women’s ability to decide or influence the decision to use contraception. The study also suggests that concealing contraception raises the woman’s chances of influencing contraception use. Other studies, such as (4), suggested that concealing contraception may indeed increase contraception use in the short run. In the long run, concealing contraception may have adverse effects in the form of moral hazard. For example, the woman may not use contraceptives if she anticipates that doing so without her husband’s knowledge, and knowing that he is opposed, would jeopardize marital stability in the event that it is discovered (4).

## Conclusion and recommendations

The study has shown some factors that would reduce the unmet contraception needs and ultimately reduce other health complications and socioeconomic burdens that come with not utilizing contraception. The study has, most importantly, built on the known knowledge by analyzing the dynamics behind the factors that influence women’s ability to decide or influence the decision to use contraception at a household level. Based on the findings, it is evident that daily household decisions play a role in arming the woman with the ability to make or influence the decision to use contraception. Consequently, if women are empowered socioeconomically and gender-wise, women will increase their ability to influence decisions to use contraception (24, 25). Nevertheless, more studies are required to investigate why uneducated women have a higher likelihood of deciding to use contraception than other women in Zambia.

The study revealed that the type of contraception women use impacts their decision-making ability regarding contraceptive use. Significant variations were observed between different contraception methods, particularly barrier methods like condoms, in terms of odds ratio and predicted probability differences. These differences underscore the role of contraceptive preference in a woman’s ability to influence its use (26). Women using methods like hormonal or reversible contraception showed a relatively lower ability to influence decisions, likely due to concerns about adverse effects. These negative perceptions hinder contraceptive use and decision-making (23). To address this, service providers should work on changing women’s preferences by dispelling misconceptions about certain methods. This can enhance their ability to influence contraceptive decisions, ultimately reducing unmet needs. This approach could empower more women to use contraception without requiring spousal approval (4).

Furthermore, the study could not establish evidence of preferences in the number of desired children and women’s ability to decide or influence the decision to use contraception. Therefore, more studies are warranted to investigate further differences in the number of desired children regarding women’s ability to decide or influence the decision to use contraception.

Lastly, the final model for this study performed reasonably well, with an AUC, the measure of the ability of a binary classifier to distinguish between classes, of 0.67. Nevertheless, this could be improved if other variables related to the circumstances that lead to marriage/living together and other dynamics within a marriage are included in the model. These variables could contribute to a better understanding of the women’s ability to influence the ability to make or influence the decision to use contraception. However, due to the limited availability of such data in DHSs, these variables were not explored in this study. Otherwise, future studies could explore such variables. Consequently, the DHS program may need to further invest in collecting more data if subsequent studies on the subject are to be more realistic (11).

## Data Availability

This study used deidentified, publicly available data from the Zambia 2013/2014 and 2018 DHS: https://dhsprogram.com/Countries/Country-Main.cfm?ctry_id=47&;c=Zambia.
